# Social Innovation: A Retrospective Perspective

**DOI:** 10.1007/s11024-022-09471-y

**Published:** 2022-07-15

**Authors:** Liliya Satalkina, Gerald Steiner

**Affiliations:** 1 Department for Knowledge and Communication Management, University for Continuing Education Krems, Dr.-Karl-Dorrek-Straße 30, 3500 Krems, Austria; 2 Complexity Science Hub Vienna, 1080 Vienna, Austria

**Keywords:** Social innovation, Historical overview, Sustainable development, Sustainability-related intervention, System innovation

## Abstract

During the last several decades, the concept of social innovation has been a subject of scientific and practical discourse. As an important paradigm for innovation policies, social innovation is also an object of criticism and debate. Despite a significant proliferation of literature, the rate at which social innovation is a catalyst for coping with challenges of modern societies remains unclear. The goal of the paper is to gain a better understanding of social innovation by integrating past and present views on the concept. Applying a historical overview covering the period from the 19th to the 21st century, we outline the milestones in the evolution of social innovation and distinguish seven trajectories that illustrate the commonalities in its interpretation. We consolidate the findings into a three-dimensional model that defines social innovation as an intervention that is targeted toward structural changes within a social dimension that, in terms of different functional settings (e.g., technological, business, organizational), are oriented on systemic improvements of societies. Reflecting on future avenues, we consider social innovation as an integrative part of a holistic intervention that acts across single societal dimensions and provides systemic impact for the sustainable development of societies.

## Introduction

The concept of *social innovation* has been increasingly discussed, particularly in the last several decades. According to the Scopus online database, from 2010 to 2020 the term “social innovation” was mentioned in peer-reviewed articles 25,014 times compared to approximately 2,135 from 1990 to 1999 and 724 times from 1980 to 1989. As a category within the general classification, social innovations are widely considered as those that “enhance society’s capacity to act” (Mulgan and Pulford 2011) by providing solutions for social problems and improvements for the quality of life (e.g., Dawson and Daniel 2010), as well as affecting social change (e.g., awareness patterns, social values, and norms) (Mulgan 2006; Mulgan et al. 2007; Phills et al. 2008; Murray et al. 2010; Davies 2012; Moulaert et al. 2013; Westley 2013). Contemporary evidence (see the section “21st century: State of the art”) indicates that, regardless of a significant proliferation of literature and research, a clear and shared understanding of the concept of social innovation continues to be lacking, making this category rather discussive and contradictive (e.g., van der Have and Rubalcaba 2016; Hölsgens 2016). For instance, whether social innovation should always be considered a phenomenon that produces beneficial results for entire societies remains unclear, and the extent to which it is an ambiguous phenomenon remains a question. Is social innovation always a “win-win solution” for real-world challenges (Fougère et al. 2017), or is it a “buzz word” (see Pol and Ville 2009) within policy practices? A holistic view of the multifaceted concept of social innovation is crucial for outlining its unique role and significance for the sustainable development of societies.

According to Phills et al., a social innovation is “created, adopted, and diffused in the context of a particular period in history” (2008); this underscores the importance of *historical evidence* for a better understanding of social innovations, particularly regarding prospective concerns and impacts on future generations. In that context, a holistic and systemic approach incorporates the understanding of past patterns that enhances the comprehension of present processes and creates a more precise orientation for coping with the uncertainties of the future (see, e.g., Baecker 1999). Therefore, the overarching goal of the paper is to gain a better understanding of social innovation by integrating past and present perspectives on the concept and reflecting on its future avenues within the prism of the sustainable development of societies. Accordingly, the research aims for a systematization of existing knowledge about social innovation based on a *historical overview* (incl. conceptual and intellectual history) that covers the period from the 19th to the 21st century. Applying a *qualitative literature analysis,* we collect contemporary and historical evidence by tracking the concept of social innovation back to the 19th century.

The article is organized as follows. In the “Methodology and research design” section, we describe the procedures used for the historical overview and qualitative literature analysis. In the section “21st century: State of the art,” we collect the contemporary evidence regarding the status of research in the field of social innovation. The section titled “19th and 20th centuries: Historical evidence” provides a collection of the interpretations of social innovation from that period. In “Findings from the historical overview,” we outline the core milestones in the evolution of social innovation conception and distinguish the key characteristics common to the 19th, 20th, and 21st centuries. In the “Discussion and outlook,” we offer a definition of social innovation based on past and present perspectives (i.e., contemporary and historical evidence). We further reflect on future patterns in the development of social innovation, considering it within the prism of system innovation and the sustainable development of societies.

## Methodology and Research Design

**Fig. 1:**
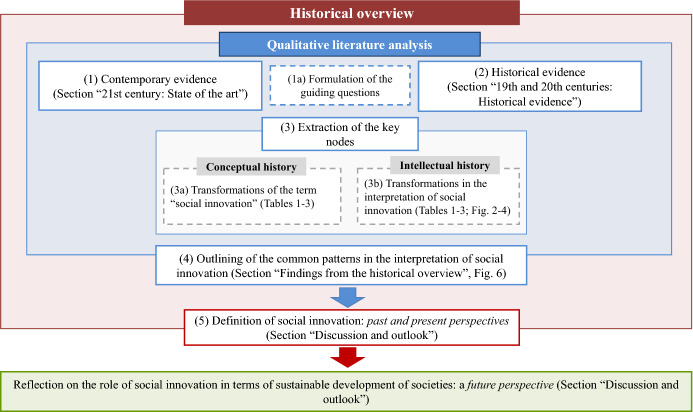
Procedure for the historical overview

To collect contemporary and historical evidence regarding the transformations of the concept of social innovation, we apply a *historical overview* that covers the period from the 19th to the 21st century and is based on the principles of *conceptual* and *intellectual history.* Collecting contemporary evidence, we aim to outline the gaps in the conception of social innovation and, thereby, specify the guiding questions for this research. Based on these guiding questions, we further collect knowledge of the past (i.e., historical evidence)*.* Accordingly, we trace the transformations of the term “social innovation” (i.e., conceptual history) as well as different approaches to its interpretation (i.e., intellectual history). The procedure for the historical overview is based on a *qualitative literature analysis* including five core steps (Fig. 1).

### *Method: Systematic Literature Review*

Since the literature analysis covered the period of three centuries, the dataset of the selected sources was very heterogenous. This provided a limitation for applying a quantitative approach for the assessment (e.g., bibliometric, meta-analysis). As a prior step an extended content analysis was needed. Therefore, we applied elements of a *systematic literature review* focusing on the qualitative structuring and synthesizing of findings, without collecting empirical evidence (see Snyder 2019; Donthu et al. 2021).

## *Search Terms and Selection Criteria*

The guiding question for the systematic literature review was: “How has the interpretation of social innovation changed over the different time periods?” As an initial step, we performed a search through the Scopus database, specifying the search algorithm with the *key term* (“social innovation”), *publication period* (2000–2019), and *type of document* (article, book chapter, book). The list of publications was sorted according to the highest number of citations. Our review did not focus on a specific disciplinary field. We selected the 100 most-cited publications and excluded those that did not include the key term “social innovation” in their titles, resulting in 48 publications. Following the guiding question, we included only those publications that concerned the general conception of social innovation inherent to the 21st century. We excluded those that focused on specific cases (e.g., country cases), concrete implications (e.g., relation to social entrepreneurship), or framework conditions (e.g., ecological framework, rural development). Finally, 13 publications were selected for full-text screening. Additionally, we performed a search through the Google Scholar database, applying the key term “social innovation” and specifying the research algorithm by the publication period (2000–2019) and relevance criteria. This allowed us to identify five additional publications relevant to our analysis. Altogether, the first dataset (i.e., dataset 1) comprised 18 sources.

To identify literature sources that were related to the 19th and 20th centuries, we screened historical research in the field of social innovation (i.e. Godin 2012a, b, 2015) and selected literature sources according to the guiding question. The criteria for selection were: (1) reference to the term “social innovation” or other terms relevant to the context (e.g., “reform,” “social innovator,” “invention”); and (2) focus on specific (often new) characteristics in the conception of social innovation. Altogether, 14 sources were selected (i.e., dataset 2).

The final list (i.e., datasets 1 and 2) comprised 32 literature sources, including books and articles in English and French, published from 1813 to 2019. The primary (archival) texts of the 19th and 20th centuries were accessed via a digital archives repository (i.e., Internet Archive^1^).

## *Screening Process*

**Fig. 2:**
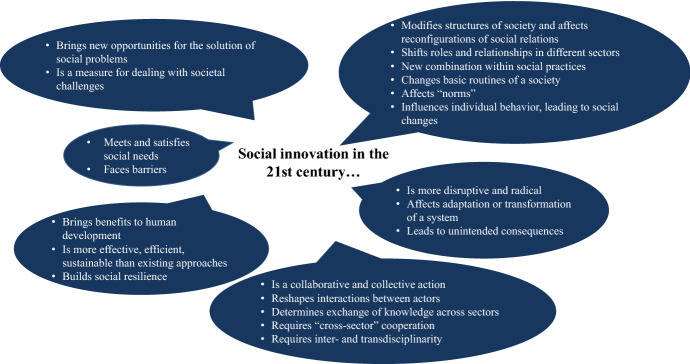
Characteristics of social innovation noted by 21st century authors

**Fig. 3:**
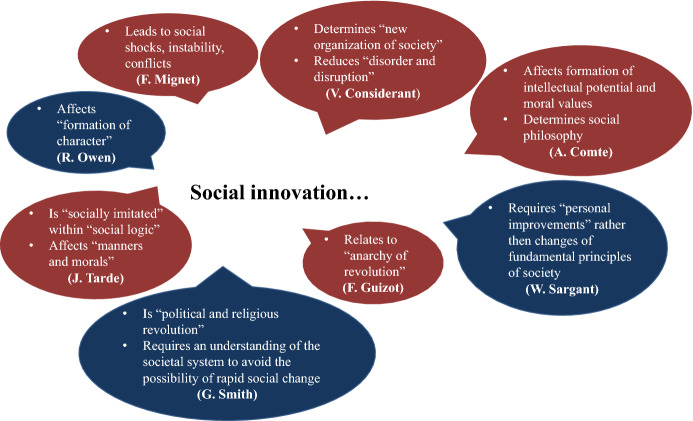
Characteristics of social innovation noted by 19th century authors

**Fig. 4:**
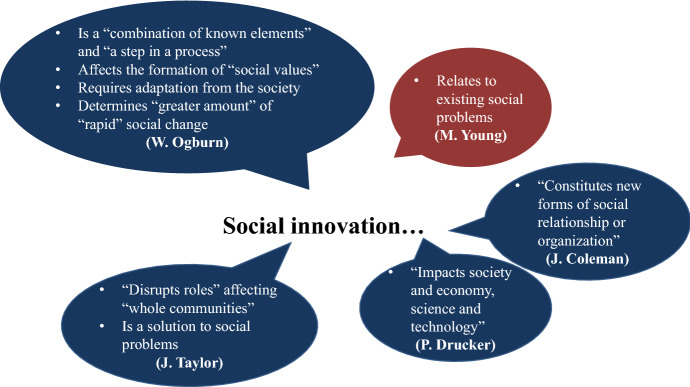
Characteristics of social innovation noted by 20th century authors

To maintain the authors’ contexts and interpretations, the extraction of key nodes was performed in three sequential steps: (1) Extraction of quotation(s) with a reference to the term “social innovation” or other relevant term; (2) Identification of the term used by the author and outlining the scopes in which it was mentioned (see Tables 1, 2, 3); and (3) Elicitation of the core characteristics of social innovation inherent to the particular century (see Figures 2, 3, 4).

## *Limitations*

We recognize several limitations of the applied methodology that, nevertheless, do not diminish the results of the qualitative analysis:

(1) Search procedure: Since the literature review covered a period of more than 200 years, not all sources could be accessed within the same database. Therefore, different approaches were applied during the identification of the relevant literature. For dataset 1, we considered only the 100 most-cited publications according to Scopus and the top relevant publications according to Google Scholar. These also reveal the potential shortcomings of citation analysis (see, e.g., Campbell et al. 2010; van der Have and Rubalcaba 2016). Dataset 2 was limited to the reference lists provided in the chosen secondary sources (no archival search was conducted).

(2) Asynchronous exclusion procedure: Within dataset 1, exclusion was conducted in two sets, i.e., by title and after the full-text screening. Exclusion by abstracts was not conducted. Within dataset 2, the selection of sources was performed during the full-text screening. Therefore, the selection procedure might be characterized by less transparency compared to, e.g., the PRISMA method (see, e.g., Satalkina and Steiner 2020) or bibliometric analysis (see, e.g., van der Have and Rubalcaba 2016).

(3) The majority of publications, particularly those of the 21st century, were related to the following disciplinary fields: “Business, Management and Accounting”, “Economics, Econometrics and Finance”, and “Arts and Humanities”.^2^ This was not determined by any selection criteria but rather related to the scope of each database (Scopus, Google Scholar). Although the analysis was extended to *Science and Technology Studies (STS),* we recognize that the research might have a certain bias toward economics and management literature.

## 21st Century: State of the Art

In the 21st century, the relation of social innovation to collective actions aimed at solving “social needs and problems” and coping with real-world challenges has become the core of the conception (e.g., Phills et al. 2008; Edwards-Schachter et al. 2012; Grimm et al. 2013). As a measure of social good (see, e.g., Ziegler 2020), social innovation attracts attention in light of ongoing discussions about the dominant role of innovation in society (“pro-innovation bias”) (see Schubert 2014; Ziegler 2020). For instance, James Phills interpreted social innovation as “a novel solution to a social problem that is more effective, efficient, sustainable, or just than existing solutions” (Phills et al. 2008: 36). Despite the significant number of scientific publications and the establishment of various research and practical institutions and initiatives^3^ for scaling social innovations (see also Schubert 2014), some scholars recognize the “fragmentariness” of the conception (van der Have and Rubalcaba 2016) and the lack of a shared understanding of the term (Hölsgens 2016; Edwards-Schachter and Wallace 2017; Fougère et al. 2017). On the one hand, the relation of social innovation to practical initiatives that aim to cope with different real-world challenges (e.g., Blue Growth Initiative by the EU) makes it an important paradigm for innovation policies (Edwards-Schachter et al. 2012; Grimm et al. 2013; van der Have and Rubalcaba 2016; Fougère et al. 2017; Schubert 2019). On the other, social innovation is an object of debate since, for some researchers, it has no concrete reference to social science and remains merely a “buzz word” (Pol and Ville 2009; Edwards-Schachter et al. 2012).

In light of institutional theory (see, e.g., P. Young 2011; Cajaiba-Santana 2014; van Wijk et al. 2019), social innovation is considered a collaborative (see, e.g., Ziegler 2017) or collective action that reshapes interactions between various actors within an institutional environment (e.g., Tracey and Stott 2017; van Wijk et al. 2019). Moulaert et al. interpreted social innovation as “the creation of new products, services, organizational structures or activities” that lead to “reconfigured social relations, and empowerment or political mobilization” (2013: 1–2). The diffusion of social innovations within institutional frameworks (e.g.,Young 2011; Hölsgens 2016) influences societal transformations (Grimm et al. 2013; Schubert 2014, 2019). Such an “intervention in ongoing social processes” is considered a core distinction of social innovations compared to, e.g., technological innovations (e.g., Schubert 2014). However, based on this principle, deriving a clear differentiation is not always possible. For instance, STS claim that technological innovations can, simultaneously, be considered social because of their impact on social practices and further complex societal transformations (e.g., Feenberg 2012, 2017a, b). In that context, the influence of technologies is also associated with potentially disruptive impacts of innovations (Cressman 2019) inherent in social innovations as well (e.g., “unintended adverse consequences” in Fougère and Meriläinen 2021). A potential concern of technologies with social purposes relates to a concept of *responsible innovations* in STS (see, e.g., Blok and Lemmens 2015; Ceicyte et al. 2021; von Schomberg and Blok 2021).

The Young Foundation (established in 1954 by British sociologist Michael Young) interpreted social innovation as “innovative activities and services that are motivated by the goal of meeting a social need and that are predominantly developed and diffused through organizations whose primary purposes are social” (Mulgan et al. 2007: 8). In Mulgan et al.’s opinion, this definition highlighted the differentiating features of social innovation compared to “business innovations, which are generally motivated by profit maximization.” However, according to Phills et al. (2008: 37), a “cross-sector” cooperation, which is an important element in the “mechanism” of social innovation, should integrate not only nonprofits and governments but also businesses. They considered new business models as a measure for meeting social needs and, thus, bringing about social innovation (e.g., “businesses are leading the way on many social issues”). Scholars have claimed that the development of business and social innovations can coincide (e.g., Edwards-Schachter et al. 2012); business innovations might have social impacts (e.g., Pol and Ville 2009), while social innovations can provide new business opportunities (e.g., Nicholls et al. 2015). Furthermore, within social practices, it is even more complicated to derive distinctions between social and business innovations (e.g., Pol and Ville 2009) since even nonprofit organizations might follow economic as well as social purposes for various reasons (e.g., competition) (see Weerawardena et al. 2021). In addition, *frugal innovations* can be examples of integration between social, business, and technological innovations, e.g., the case of smallholder farmers in Kenya (see Musona 2021). Consequently, some scholars doubt that the concept of social innovation should be understood as only a category in the classification rather than as an independent paradigm that characterizes the social aspects of any innovation activity (van der Have and Rubalcaba 2016). For instance, social innovation is defined as a “targeted new combination of social practices” (Howaldt et al. 2015) that influences individual behavior (Hölsgens 2016), potentially demonstrating the mechanisms “that result in positive social change” (Phills et al. 2008). Interconnecting social innovation with social change, Mulgan et al. (2007) identified three lenses: individuals, movements (e.g., feminism, environmentalism), and innovative organizations. They further discussed four interconnected barriers to social innovation as a social change: (1) efficiency (i.e., resistance related to the “worsened performance” of reforms compared to the previous experience); (2) interests (i.e., the risks related to changes seem more significant than the potential benefits); (3) minds (i.e., the social system is “solidified” in its “assumptions, values, and norms”); and (4) relationships (i.e., personal relations within the system) (Mulgan et al. 2007). In that context, the diffusion of social innovation might be different compared to other types of innovation (e.g., Westley and Antadze 2010; Hölsgens 2016).

The generation of transdisciplinary and interdisciplinary knowledge about social innovations, with respect to resilience and sustainability, is one of the core directions of the Waterloo Institute for Social Innovation and Resilience (WISIR). Westley (2008) considered social innovation in the scope of a dynamic that increases the resilience of societal systems and provides new ideas, practices, and policies or resource flows for the solution of social problems. She suggested that understanding social innovation requires an understanding of how a novel solution “enters and transforms,” leading to complex interactions and determining new roles for “human agency” (Westley and Antadze 2010). She further noted that, in terms of system resilience, innovations either “allow for adaptation” or “have potential to transform the system,” being “more disruptive and radical,” but that the special role of social innovation is to enable an understanding of how social systems “adapt or transform” (Westley 2013). Social innovation is not a single solution but “part of a process that builds social resilience and allows complex systems to change” (Westley and Antadze 2010). An important characteristic of social innovations is that they “profoundly change the basic routines, resource and authority flows, or beliefs of the social system in which the innovation occurs” (Westley and Antadze 2010).

The state of the art indicates that, in the 21st century, international interest in social innovation as both a scientific and applied category is continuously emerging (Table 1), while various studies have revealed different characteristics of the concept (Fig. 2). Based on the contemporary evidence, it is possible to identify certain gaps in the conception where shared understanding among scholars is lacking. Accordingly, we outline three guiding questions for further analysis:

- *GQ1:* Is social innovation just a “buzz word” or does it have significance?
- *GQ2:* Does social innovation have its own niche in the classification?
- *GQ3:* Does social innovation have the potential to identify paths toward sustainable development?

**Table 1:**
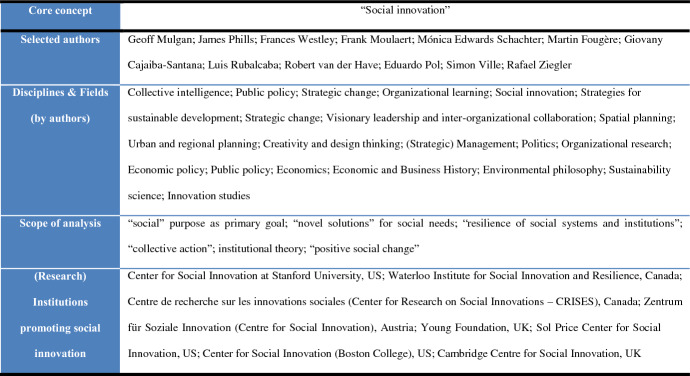
Social innovation ideas in the 21st century

## 19th and 20th Centuries: Historical Evidence

### 19th Century: Utopianism, the French Revolution, and Social Imitation

During the 19th century, political economists, sociologists, and historians reflected on the nature of social changes and their impacts on societies. The British political economist and educational reformer William Lucas Sargant reflected on the idea that social change had to “revolt against injustice” and improve the “destiny of the working classes” based on the examples of French utopians (e.g., Saint-Simon, Fourier, Louis Blanc, Proudhon, and Emile de Girardin). He critically analyzed their “remarkable” results. For Sargant, the basic “machinery of individual capitalist and labourer” with its political and economic principles shouldn’t be changed. He considered personal “improvement” the most important factor for bettering society (see Sargant 1858: 463).

The British historian, journalist, and controversialist Goldwin Smith associated social innovation with “the political and religious revolution which fills the civilized world” (Smith 1883: 4). He criticized socialists, communists, and nihilists for their doctrines of social change, particularly those related to the problems of inequality between social classes or the distribution of wealth. He argued that such doctrines don’t “reconstruct” but rather “destroy” social order with its institutions and established morality (Smith 1883: 3). Smith suggested that ideas for solving social problems such as poverty and the inequity of wealth distribution may be reasonable but could not be achieved with extreme and radical methods that lead to “violent shocks” of the social system (e.g., French Revolution) (Smith 1883: 3). On the contrary, he believed that the process required an understanding of the social system leading to the “experiment which may hold out a reasonable hope of putting an end to poverty” (Smith 1883: 4). Suggesting that rapid social change may result in chaotic and disruptive impacts, Smith considered it impossible to find solutions for all social inequalities (e.g., health, intellectual power) (Smith 1883: 4–5).

Reflecting on such social changes and their potential consequences, 19th century analysts sometimes associated “social innovators” with those who inspired and followed revolutionary events with, at some point, controversial consequences. French historian François Mignet, whose works had a strong influence on French historians’ studies, analyzed how “social change” may controversially affect the existing social order and social relations, describing the revolutionary situation in Lyon in 1790. He emphasized that the actions of innovators, declared to be improvements to the socioeconomic order, might, on the contrary, lead to even more negative social shocks. He used the term “pacific innovation,” meaning that innovation “must not be contested”; otherwise, it leads to social instability and conflicts (Mignet 1846: 248). Mignet differentiated between “wise and temperate reformers” and “extreme and inflexible innovators” (Mignet 1846: 248).

François Guizot, French politician, historian, and conservative constitutional monarchist, used the term social innovators *(novateurs sociaux),* relating it to the “anarchy” of revolution (Guizot 1859: 208). Similar to Mignet, he distinguished between “radical reformers” and “systematic innovators” and highlighted their different impacts on the established institutional order (Guizot 1862: 166).

The French philosopher Auguste Comte, considered a founder of sociology and positivism, stressed the important role of social innovation in the formation of intellectual potential and moral values (Comte 1864: 259). Comte used the term social innovation *(innovation sociale)* in relation to Catholicism, pointing to its role in the development of moral and intellectual potential*,* with further impacts on the formation of social philosophy *(philosophie sociale)* and social values *(valeur sociale)* (Comte 1864: 308).

In contrast to Smith and Sargant, who criticized the Utopian Movement, French socialist and political scientist Victor-Prosper Considérant highlighted the positive effect of social changes on the “new organization” of society *(organisation nouvelle).* As a leader of French Fourierist Utopianism (following the death of Charles Fourier in 1837), he applied the term social innovation *(innovation sociale)* in the following context: “We feel that society is ill-at-ease, we admit that it needs the new organization: the current state of affairs causes disorder over disorder, disruption over disruption, and all that obviously can only be stopped by a social innovation” (Considerant 1837: 312).

As the 19th century also witnessed the Industrial Revolution, ideas related to social changes found support, to some extent, among people involved in industrial life. Their ideas about social reforms concerned social transformations based on new industrial policies. The British manufacturer Robert Owen, one of the most influential utopian socialists of the 19th century, was well-known for his social and industrial welfare programs in the New Lanark cotton mills in Lanarkshire (Scotland). Earlier than Considérant, Owen developed the idea of the “rational training” of the population, which, he believed, could influence the formation of individual character that could be transferred to “any community, even to the world at large.” He suggested that an important condition for the formation of character was “rational reform” (or “reform in the training and in management of the poor”) that was related to “modern improvements of education.” He emphasized the importance of an educational system that would affect the formation of individual character (Owen 1813: 61) and pointed to the critical role of parents in education and character formation.

Significant input into the development of innovation theory by the end of the 19th century came from French sociologist and criminologist Gabriel Tarde. He proposed a theory that explained the repetition of social life on principles of “imitation,” which he called “the cause of all social likeness” (Tarde 1903: 37). Tarde suggested that any innovation is “socially imitated” because all social tendencies are regulated by “social logic” and are the results of imitation, and that “nothing in history is self-creative” (Tarde 1903: 150). He pointed to the role of the mind and derived the interrelatedness between the individual and the national “mind” (Tarde 1903: 151). He described the effects of inventions on the formation of “manners and morals” and on the further formation of modern civilization (Tarde 1903: 344–345). In his research, Tarde stated: “… it is easy, moreover, to show that our innovations are, for the most part, combinations of previous examples, and that they remain outside of the social life so long as they are not imitated” (Tarde 1899: 40). Tarde’s ideas had a strong influence on the development of innovation theory in the 20th century (e.g., distinction between invention and innovation and innovation as rearranged “combinations” by J.A. Schumpeter). Table 2 and Fig. 3 represent an overview of the main chronological milestones, terminology, and characteristics related to the development of social innovation conception in the 19th century.

**Table 2:**
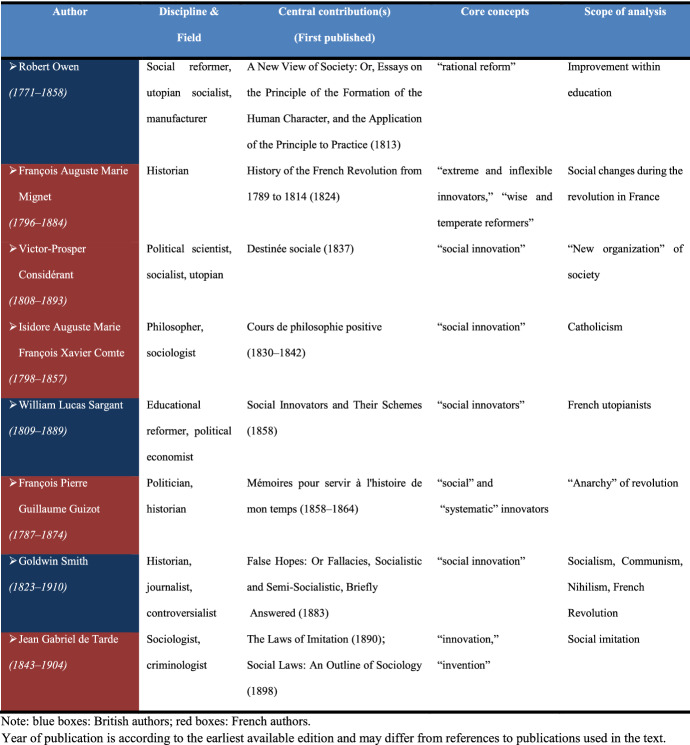
Chronological milestones of social innovation ideas in the 19th century

### 20th Century: Management and Interdisciplinarity

Gabriel Tarde’s idea that innovations are “combinations of previous examples” was further developed in works besides those of Schumpeter. The American sociologist William Fielding Ogburn interpreted “mechanical invention” and “social invention” as “a combination of known elements into a new element” (Ogburn and Nimkoff 1946: 790) and “a step in a process rather than the entire creation of something new” (Ogburn 1926: 228). He explained this with the example of the telegraph, which combines a battery, electromagnet, and wire (Ogburn and Nimkoff 1946). Further extending Tarde’s ideas about the role of the individual mind in “imitation,” Ogburn noted that “mental ability” is one of the key factors for invention (Ogburn 1926: 228). He demonstrated more distinctly the connections between inventions (“mechanical” or “social”) and the formation of “social valuations,” interlinking both types of inventions by “the nature of the influence of inventions on society” (Ogburn and Gilfillan 1933: 124). Similar to several 19th century thinkers, Ogburn pointed out that the social impacts of inventions (and relevant social changes) require adaptations on the part of society and may depend on how widely an invention is adopted (Ogburn and Gilfillan 1933: 125). According to Ogburn, the effects of social inventions on culture determine “not only a greater amount of social change but a more rapid social change” (Ogburn 1923: 331).

As was the case with reformers of the 19th century (see Owen 1813), social innovation in the 20th century was related to practical initiatives. One of the most well-known innovators was the British lawyer, sociologist, and social reformer Michael Dunlop Young. Although he rarely mentioned the terms innovation or social innovation in his publications (e.g., *Family and Kinship in East London,* 1957, and *Social Scientist as Innovator,* 1983), he was famous for “innovations in the world of action” (Young 1983: 255), such as the Consumers’ Association, the Advisory Center for Education, the International Extension College, and the Mutual Aid Center. A significant number of his initiatives were related to education; one of the best-known is the Open University, a “combination of correspondence, broadcasting and face-to-face teaching” (Young 1983: 71). Young brought attention to existing social problems, emphasizing the need for certain changes or promoting his initiatives and ideas, sometimes in satiric form (see e.g., Young 1983: 254). According to Young, the new university should include a “second shift of teachers,” meaning that, included among university teachers, there should be people from science and practice (“They will have to come from qualified people doing other things – mainly from [sic] scientists and others working in industry…”) (Young 1983: 65).

The term social invention in the context of social relations was also discussed in the work of American sociologist James S. Coleman. Coleman distinguished between social inventions that might “constitute fundamentally new forms of social relationship, or new forms of organization” and those that “constitute no new form of organization, but instead use existing forms for an organization directed toward new goals” (e.g., labor units) (Coleman 1970: 163). Coleman considered “social organization based on roles rather than on persons” as a change in social relations and, therefore, social invention (Coleman 1970). As an example, he discussed bureaucracy, which is based on “roles” and “relationships between roles,” while persons “fill the roles.” Analyzing the “role-based mode of organization” from a historical perspective (Roman society, early Greece, the Middle Ages, etc.), Coleman considered the modern organization (corporation) as “one of the most important social inventions in history” (Coleman 1990: 170). He mentioned that social inventions happen because new conditions “make them possible” or “create a use for them” (Coleman 1970: 163).

Significant input into the development of the social innovation concept was added in the second half of the 20th century by Peter F. Drucker, the Austrian-American economist who was a leader in the advancement of management education. Drucker wrote: “Social innovations – few of them owing anything to science or technology – may have had even profounder impacts on society and economy, and indeed profound impacts even on science and technology themselves” (Drucker 1987: 29). He further defined management as the “agent of social innovation” and considered five examples of social innovation of that time: the research lab, the Eurodollar and commercial paper, mass and mass movement, the farm agent, and management (Drucker 1987).

Significant input into the development of the conception of social innovation in the 20th century came from James B. Taylor, with his article “Introducing Social Innovation” (an edited version of a paper read at the American Psychological Association Meeting, August 31, 1968). Although the article referred to social innovation, Taylor also applied the term “social invention,” for which he distinguished between two problematic points. The first is that social inventions can “disrupt complex and valued roles, identities, and skills” (e.g., an innovative school for children from different social classes or with different health problems) (Taylor 1970: 70). The second point is the “problem of interdisciplinary cooperation” (i.e., the difficulties encountered when people from different disciplines attempt to work together). In the article, Taylor does not define social innovation but relates the understanding of that concept to a project of psychological rehabilitation for people with low incomes (Taylor 1970: 71), which could serve as a “model for social innovation.” In that context, the understanding of social innovation provided by Taylor shares similarities with the modern interpretation of social innovation as a “solution to social needs.”

The results of our analysis indicate that, in the 20th century, the greatest impact on the development of social innovation came from American researchers, mostly sociologists. Table 3 displays the main chronological milestones, terminology, and characteristics related to the development of social innovation conception in the 20th century. The main characteristics of social innovation discussed by authors during that period are shown in Fig. 4.

**Table 3:**
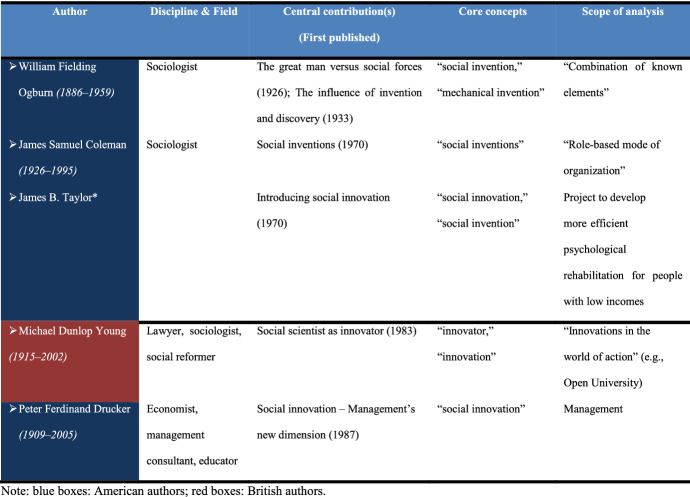
Chronological milestones of social innovation ideas in the 20th century

## Findings from the Historical Overview

### Evolution of the Social Innovation Conception from the 19th to the 21st Century

The historical overview showed that social innovation is a complex phenomenon based widely on a philosophy formed through analytical reflections and interdisciplinary contributions, with respect to different time frames and social events. In the 19th century, the terms social innovation and social innovator lacked a distinct conceptual background. Frequently applied by philosophers and historians of the time, they were rather philosophical concepts used in reflections on social injustice and social changes. On the one hand, the understanding of social problems determined ideas about changes or reforms (e.g., R. Owen). On the other, understanding potential imbalances that radical innovation might bring to a societal system determined the critical vision and distinction between different ways of innovating (“wise and temperate reforms” or “extreme and inflexible”) (e.g., F. Mignet, F. Guizot, G. Smith). It is difficult to conclude that the interpretation of social innovation was negative, even among those thinkers who were somewhat critical. Criticism was often determined by the association of social innovation with several doctrines considered radical for the time (e.g., socialistic, communistic, or nihilistic). While some thinkers considered social innovation through the prism of revolution (e.g., F. Mignet, F. Guizot), directly associating it with “violent shocks”, others saw it as a solution for social “disruption” (e.g., V. Considérant). Nevertheless, the 19th century became an important period for the formation of social innovation conception, relating it to the development of social philosophy and social values (e.g., A. Comte). At the end of the century, with the works of Tarde, social innovation began to transform from an abstract philosophical category toward a scientific paradigm.

In the 20th century, philosophical reflections on social changes typical of the previous century were put into frames of sociology and management. Within “social invention” and “social innovation,” researchers formulated the distinct elements of scientific conception: factors (by W. Ogburn), drivers (“incentives” by W. Ogburn or “encouraging conditions” by J. Coleman), and main actors (“private sector” by P. Drucker). Thereby, in this century, social innovation was transformed from an abstract philosophical construct to a defined term (e.g., the definition provided by P. Drucker) that was used in relation to applied practical models (e.g., an interdisciplinary project in Kansas).

**Fig. 5:**
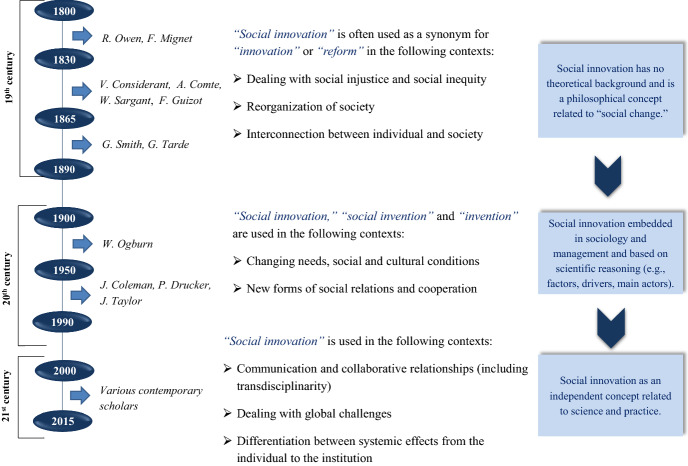
Evolution of the social innovation conception from the 19th to the 21st century

In the 21st century, social innovation as a scientific as well as (policy-related) applied category continues to evolve. Various studies have revealed different facets of social innovation, but the commonly noted characteristic is the relation of social innovation to the solution of social problems (“social needs and problems” per J. Phills et al.; “long-standing global problems” per G. Moulgan et al.; “social problem areas” per F. Westley; “social needs” per G. Mulgan, etc.). Figure 5 depicts the core milestones in the evolution of the social innovation concept from the 19th to 21st centuries.

### Trajectories of Social Innovation Within the Historical Traces

**Fig. 6:**
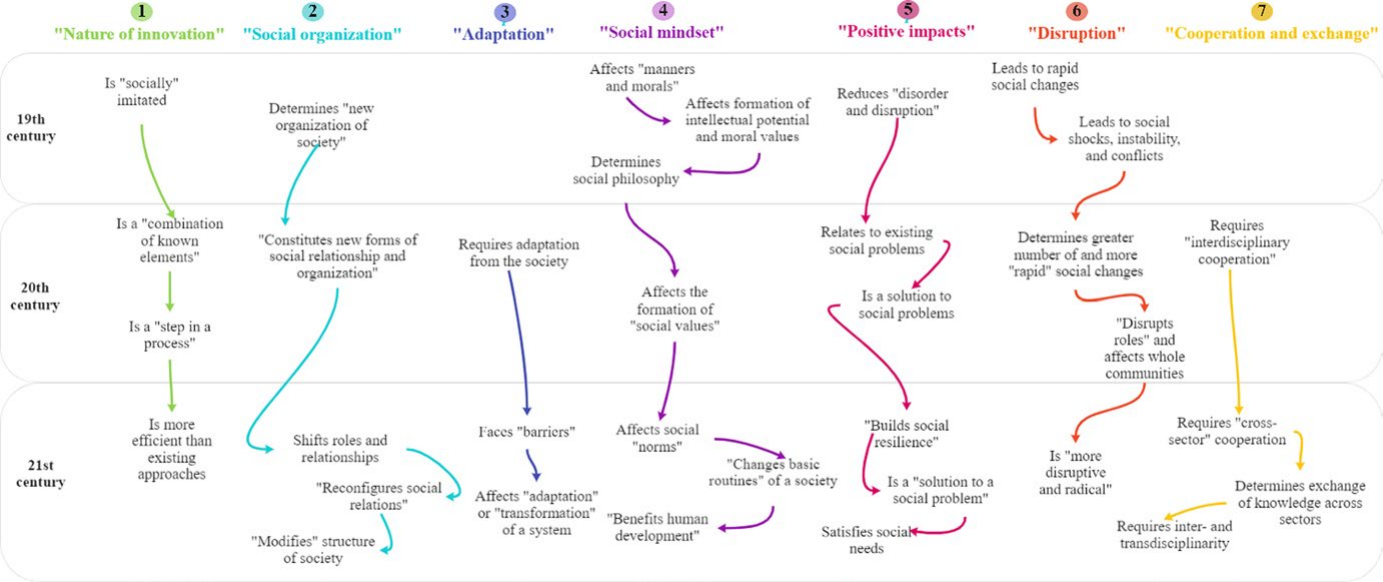
Common patterns in the interpretation of social innovation from the 19th to the 21st century

The main characteristics elicited during the historical overview (see Figs. 2, 3, 4) allowed for distinguishing seven trajectories that illustrate the common patterns in the interpretation of social innovation during three centuries: Nature of innovation, Social organization, Adaptation, Social mindset, Positive impacts, Disruption, Cooperation and exchange (Fig. 6).

*Trajectory 1* shows that, from the 19th century on, social innovation has been associated with various forms of inventions that were integrated into social practice and seen as improvements of existing procedures. The diverse implications of social innovations have been related to new combinations of “previous examples” (J. Tarde in the 19th century) or “known elements” (W. Ogburn in the 20th century) and considered more efficient compared to “traditional approaches” (F. Moulaert et al.). These improvements affect the existing relations between various agents in a social system (*Trajectory 2*). Particularly, the generation and further implementation of social innovations expand their multidimensional impacts, fostering “new forms of social relations” (J. Coleman), shifting existing “roles within different sectors” (J. Phils et al.), and affecting the structure of societies (“new organization,” V. Considérant; “modified structure,” F. Moulaert et al.). Such a “reconfiguration of social relations” requires adaptations of a social system (*Trajectory 3*) and might be confronted with “barriers” that are either individual-related (i.e., patterns of mentality, personal expectations and interests) or institution-based (e.g., barriers within bureaucratic organizations, policy interests). The adaptations of the social system also involve changes in social mindsets (*Trajectory 4*) that, on the individual level, lead to the transformation of “social values” (e.g., “manners and morals”) influencing intellectual potential, moral values, and “human development.”

Social innovation is considered a measure for improving societies (“measure against disorder and disruption”) that offers solutions to societal problems, challenges, and needs (*Trajectory 5*). However, *Trajectory 6* illustrates potential disruptive and radical changes generated by social innovation that result in complexity for understanding its consequences, particularly with respect to all involved agents and within different time frames (concerning future generations as well). This complexity requires the activation of cross-sector knowledge exchanges and new forms of inter- and transdisciplinary collaboration (*Trajectory 7*).

## Discussion and Outlook

### Definition of Social Innovation: Past and Present Perspective

The seven trajectories (see Fig. 6) demonstrate that, within different historical settings, social innovation has been commonly considered a component in various social changes. On their own, the trajectories do not provide an obvious definition of social innovation as a self-reliant category. However, they illustrate the common characteristics that, for three centuries, have been considered intrinsic and, at some point, foundational to the concept. Based on the collected contemporary and historical evidence, we can define *social innovation* as an intervention that is targeted toward structural changes within a social dimension that, in terms of different functional settings (e.g., technological, business, organizational), are oriented on systemic improvements of societies. Under the “social dimension” we understand the system of social structures in which individuals continuously interact (Weber 1947; Lenski 1984, 2005) create and exchange knowledge, fostering changes and aiming for improvements (Nolan and Lenski 2011; Scholz 2011). Social structures include social organization (e.g., social order, classes, roles, positions, primary groups, order of knowledge) and social institutions (e.g., religion, education) (Stehr and Weingart 2000; Lenski 2005; Scholz 2011).

**Fig. 7:**
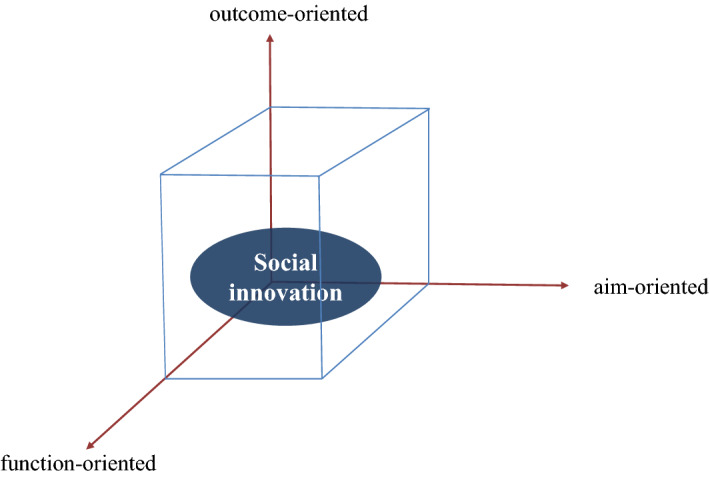
3-D model of social innovation

The proposed definition frames social innovation within a three-dimensional model, depicting it as a function-, aim-, and outcome-oriented intervention (Fig. 7).

From the 20th century, when social innovation becomes a distinct term, the concept is also explicitly associated with novel accomplishments in, e.g., technologies, business spheres, and organizational structures. It can be said that social innovation has been interconnected with other types of innovations by designating their implementation into social practices (see, e.g., Ogburn’s reflections on the interlinkages between mechanical and social inventions) and conveying their functional characteristics (i.e., function-oriented). For example, social enterprise, mentioned by Nicholls et al. as a “subset of social innovation,” can itself, at some point, be considered a social innovation related to new business model(s) (2015). However, a qualitatively different characteristic of social innovation within the normative classification can be identified if we consider *Trajectories 2, 4,* and *5* as root causes rather than consequences of such intervention. This means that, compared to other interventions, social innovation is specifically targeted (i.e., aim-oriented) toward systemic changes within a social dimension in order to enhance societal improvements (i.e., outcome-oriented) (see e.g., Considérant 1837; Phills et al. 2008; Taylor 1970; Westley 2008). In other words, the changes in the system of social structures are seen as the core aim of the intervention and a prerequisite for enhancing societal betterments. For instance, Moulaert et al. (2013: 13) viewed social innovation as a “label” for defining the development of society (“how its structures are modified, its ethical norms revisited, etc.”). However, this does not eliminate the concern about potential disruptions (*Trajectory 6*). Evidence for this can be found in the ideas of scholars of the 19th century who claimed that “improvements” to societies cannot be achieved rapidly (“wise and temperate reforms” by F. Mignet). They should begin on the individual level (e.g., “personal improvement” by W. Sargant), gradually changing and possibly improving social relations.

### Reflection on Future Perspectives of Social Innovation

Both contemporary and historical evidence show that different interpretations of social innovation have conformed to emerging societal priorities that required changes in social relations, e.g., social reforms in the 19th century and new forms of organizations and collaboration in the 20th century. The turbulence of modern societies requires interventions that will enhance their sustainable development (Laws et al. 2004) and enable them to cope with uncertainties. However, this requires systemic considerations of not only different time boundaries (i.e., “inter- and intragenerational justice”) but also different dimensions, e.g., of coupled human–environment systems (Weisz et al. 2001; Haberl et al. 2011; Scholz 2011). According to an OECD report, discrete policy interventions are not sufficient for coping with real-world challenges that are systemic by nature (OECD 2012). The complexity of real-world challenges (e.g., climate change) requires holistic innovations since segregated and rapid interventions might instead lead to disruptions (see perspectives of the 19th century). Therefore, in the 21st century, social innovation should be an intervention for systemic improvements of societies and should generate a *higher level of their sustainability*. Based on this concern, an important question arises: “*How can social innovation be targeted to identify sustainable implications?*”

Successful innovations create value based on understanding “users’ needs” (see, e.g., von Hippel 2007). This new value is not always initially required but is accepted by users as soon as they start experiencing it (see Verganti 2009). According to Phills (2008), social innovation is a solution “for which the value created accrues primarily to society as a whole rather than private individuals” (Phills et al. 2008: 36). Yet, how can the needs of the whole society be outlined without creating contradictions among its various agents (e.g., individuals, groups, communities)? As a sustainable intervention, a social innovation should address the interests of various individual stakeholders. Thus, the identification of needs for social innovation (Mulgan et al. 2007: 9) might be entangled since, apart from a normative view on its exclusive benefits, social innovation might in fact be perceived differently from the perspectives of various stakeholders (see, e.g., Nicholls et al. 2015). This provides evidence of a particular role for “collaborative capabilities” (e.g., Steiner et al. 2015; Steiner 2018) for the development of *sustainability-related interventions*. According to Drucker (1987), in the 19th century, social innovation resulted from the actions of governments rather than those of the private sector, while in the 20th century, it became the scope of the “private, non-governmental sector.” For managing the complexities of the 21st century, collaboration and exchange between various agents (e.g., science, market, policy, publicity) are critical (Jasanoff 2003; Jasanoff and Kim 2015; Pfotenhauer and Jasanoff 2017; Felt 2020). Ill-defined real-world challenges require nondiscrete interventions that act across single societal dimensions (see also coupled human–environment systems in Scholz 2011) and connect agents from policy, business, science, etc. (see, e.g., OECD 2012). Can such interventions be related to one specific type of innovation? Or should they, instead, integrate a *portfolio of different innovations* that simultaneously affect different dimensions by merging, e.g., process, product, and structural innovations, as well as, e.g., business, technology, and social innovations? This aligns with the paradigm of a *system innovation* that integrates social innovations and determines a cross-boundary collaboration between involved stakeholders for coping with complex systemic problems (OECD 2015). This raises an important question: *“How can a toolkit needed for the generation of sustainability-related interventions be developed?”* According to the OECD (2015), system innovation policies require sophisticated analytical tools that facilitate the understanding of causal relations within systemic problems, including relations between involved stakeholders. Therefore, mutual learning between various agents (i.e., from science and practice) becomes a critical prerequisite for the joint system understanding of complex, ill-defined societal challenges (see Scholz and Steiner 2015a). For instance, “collective intelligence” is recognized not only as essential for “democratizing” the generation of innovations (see von Hippel 2005) but also for establishing partnerships among various parties (see von Hippel 2007). This provides evidence for the importance of inter- and, particularly, transdisciplinary collaboration as parts of a toolkit for integrating different forms of epistemics (Scholz and Steiner 2015b) and designing systemic sustainability-related interventions.

## Conclusions

The existing research offers clear evidence of the multifaceted character of social innovation, defining it within complexity and sustainability perspectives and theories (e.g., McGowan et al. 2017; Steiner 2018) as well as referring to its systemic impact on societies (Olsson et al. 2017). As a dimension within an innovation philosophy, the conception of social innovation followed at least 200 years of marginal development (see Fig. 5). It has been embedded in the prism of sociocultural tendencies (e.g., 19th century), technological achievements (e.g., conceptions of “mechanical” and “social” inventions in the 20th century), and societal challenges (e.g., ideas of “long-standing global problems” in the 21st century).

The results form a foundation for extended research in the field of innovation and its systematic impact on the sustainable development of societies. An important research avenue relates to the development of a holistic view of a system innovation as a *“portfolio of innovations”* that can be a driver for sustainable development. An understanding of potential tools, including mutual learning between various agents, is a vital condition for developing and targeting such sustainability-related interventions. A further research avenue relates to extended *cross-boundary historical analysis* (for different geographic, cultural, and political boundaries) embedded within the *systematic research framework.* A historical analysis is important for understanding the practices of the past and their impacts in order to build scenarios for the future (see e.g., Young 1983). Understanding the core patterns in the development of innovation systems from the perspective of different models of innovative societies may become critical for the development of such scenarios and comprehending their multidimensional impacts. This might provide a basis for understanding the sustainable influences of innovations (see Pfotenhauer and Jasanoff 2017) as well as potential vulnerabilities that might be inherent to (global) societies. An example here may relate to the various and widespread impacts of COVID-19 within specific socioeconomic systems as well as its integrative impacts on entire societies. Furthermore, such a systematic and integrative understanding of social innovation may contribute to the effects of specific innovation initiatives for coping with global challenges (e.g., SDGs, Grand Challenges).

## Data Availability

Not applicable.

## Footnotes

1 https://archive.org/.

2 Subject areas are provided according to the Scopus classification.

3 E.g., the EU’s BENISI project "*Building a European Network of Incubators for Social Innovation*".
